# Global English-language-dominated discourse on artificial intelligence in healthcare: a three-year longitudinal analysis of the #AIinHealthcare movement on X

**DOI:** 10.3389/fdgth.2026.1795488

**Published:** 2026-04-17

**Authors:** Thomas Wochele-Thoma, Thadiyan Parambil Ijinu, Sreejith Pongillyathundi Sasidharan, Anoop Manakkadan, Lathikakumariamma Sahadevakurup Shine, Neenthamadathil Mohandas Krishnakumar, Selvaraj Indira Aruna, Nagarjuna Pasupuleti, Thomas Aswany, Divakaran Chandramathi Deepthi, Zilin Ma, Yining Hua, Michał Ławiński, Olena Litvinova, Maria Kletecka-Pulker, Atanas G. Atanasov

**Affiliations:** 1 Ludwig Boltzmann Institute Digital Health and Patient Safety, Medical University of Vienna, Vienna, Austria; 2 BioX Intelligence Hub, Naturae Scientific, Kerala University-Business Innovation and Incubation Centre, Thiruvananthapuram, Kerala, India; 3 The National Society of Ethnopharmacology, Thiruvananthapuram, Kerala, India; 4 Nutraceuticals-India Consortium, Naturae Science Foundation, Thiruvananthapuram, Kerala, India; 5 Academy of Bharatiya Knowledge Systems and Traditions, Thiruvananthapuram, Kerala, India; 6 Multidisciplinary Research Unit (Department of Health Research, MoH&FWD, GoI), Government Medical College, Thiruvananthapuram, Kerala, India; 7 HealthTech Intelligence and Innovation Hub, Skysmile Technologies, Thiruvananthapuram, Kerala, India; 8 Department of Biosciences, Rajagiri College of Social Sciences (Autonomous), Ernakulam, Kerala, India; 9 Department of Botany and Computational Biology, St. Aloysius College, Thrissur, Kerala, India; 10 Medomas Healthcare, Thiruvananthapuram, Kerala, India; 11 Department of Biotechnology, SCMS School of Technology and Management, Ernakulam, Kerala, India; 12 Intelligent Interactive Systems Group, Harvard School of Engineering and Applied Sciences, Harvard University, Allston, MA, United States; 13 Department of Epidemiology, T.H. Chan School of Public Health, Harvard University, Boston, MA, United States; 14 Institute of Genetics and Animal Biotechnology of the Polish Academy of SciencesMagdalenka, Poland; 15 Department of General Surgery, Gastroenterology and Oncology, Medical University of Warsaw, Warsaw, Poland; 16 Department of Management, Marketing and Quality Assurance in Pharmacy, National University of Pharmacy of the Ministry of Health of Ukraine, Kharkiv, Ukraine; 17 Patient Safety and Digital Health (PaDiH) Group, Danube Private University, Krems-Stein, Austria; 18 Department of Biochemistry, Saveetha Medical College and Hospital, Saveetha Institute of Medical and Technical Sciences, Chennai, Tamil Nadu, India

**Keywords:** clinical applications, data ethics, digital health, generative AI, healthcare, patient safety, responsible AI, social media

## Abstract

**Background:**

Social media platforms facilitate global discourse on the application of artificial intelligence (AI) in healthcare. Nevertheless, there is a paucity of longitudinal analyses of digitally mediated discussions.

**Objective:**

To investigate the evolution of global English-language-dominated discourse on #AIinHealthcare over a three-year period on X (formerly Twitter).

**Methods:**

Using Fedica analytics, we analysed 57,880 tweets by 17,991 distinct users across 141 countries from 1 November 2022 to 1 November 2025. This analysis focused on English-language-dominant discourse around #AIinHealthcare (96.9% English), acknowledging hashtag-specific selection bias and linguistic limitations. This study used publicly available anonymised data and followed the ethical guidelines for social media research.

**Results:**

The #AIinHealthcare garnered 39.2 million impressions, with significant contributions from high-income countries, notably the United States (40.7%) and Canada (21.0%), as well as India (13.4%; a rapidly expanding economy), collectively accounting for 75.1% of tweets and reflecting hashtag-specific, geographically concentrated engagement. This peaked in mid-2023 and stabilized lower by mid-2025. English was the predominant language of the discourse (96.9%). The community consisted of 74.9% grassroots users with fewer than 1,000 followers, suggesting genuine participation beyond elite influencers. Total engagement reached 72,625 interactions, primarily passive, comprising 68.1% likes, 19.4% retweets, 10.3% replies, and 2.1% quote tweets. Hashtag co-occurrence patterns, supported by qualitative inspection of exemplar tweets, indicated majorly five distinct clusters: foundational technical topics (#GenerativeAI, #ChatGPT, #LLMs) peaked after November 2022; clinical application themes emerged across disease-specific specialties (#Oncology, #Cardiology, #MentalHealth); healthcare implementation themes addressed practical integration (#DigitalHealth, #Telemedicine, #EHR); governance and ethics themes gained prominence (#ResponsibleAI, #AIEthics, #ExplainableAI, #DataPrivacy); and professional integration themes fostered learning communities (#MedTwitter, #MedicalEducation). Sentiment was predominantly neutral (95%), with positive (3%) and negative (2%). Monthly tweets peaked in mid-2023 at 1,600–1,800 before declining to 750–900 per month by June 2025. High-engagement content linked AI to practical applications, governmental initiatives, and clinical breakthroughs.

**Conclusion:**

English-language-dominated discourse around #AIinHealthcare reveals hashtag-specific maturation from technical enthusiasm to governance and implementation focus. However, platform access restrictions in countries such as China and Russia may skew geographic representation. Disparities in sustainability discourse remain prevalent.

## 1 Introduction

In recent years, the rapid development of artificial intelligence (AI) in healthcare has significantly transformed professional discourse and clinical practice. While this progress has generated optimism regarding more accurate, efficient, and equitable healthcare systems, it has simultaneously exposed substantial ethical, regulatory, and social challenges, including algorithmic bias, transparency, patient safety, data governance, and accountability (1–11). AI-based tools, such as deep learning algorithms in radiology and pathology, as well as predictive models for sepsis and readmission, are increasingly being integrated into clinical decision-making, population health management, and healthcare system operations (12, 13). As AI moves from the experimental phase to routine practice, concerns regarding ethics, transparency, and safety continue to grow (14–16). In addition, AI in healthcare raises fundamental questions about trust and the doctor-patient relationship (17). As AI contributes to clinical decision-making, debates have emerged regarding responsibility, patient perceptions, and transparency. AI represents a sociotechnical transformation that reshapes the relationships between clinicians, patients, and institutions (18).

A critical turning point in the development of the discourse was the emergence of large generative models, primarily ChatGPT, launched in November 2022, which accelerated technological integration and significantly expanded the scale and intensity of public discussions of AI in medicine (19). These discussions are increasingly moving beyond academic journals and policy documents and taking shape in real time on digital platforms. Social media, particularly X, has become an important space where doctors, researchers, patients, technologists, and policymakers from around the world discuss the benefits and risks of AI in healthcare, share empirical data, and shape narratives that can influence research priorities, policy decisions, and public trust (20, 21). In this context, hashtags such as #AIinHealthcare function as socio-technical tools that not only structure information but also bring participants together around this specific tag, forming transnational discursive communities (22–24). Prior studies have shown that analyzing hashtag-driven discussions allows us to track changes in public and professional opinions, identify key participants, and assess the dynamics of discourse in response to technological and regulatory shifts (25–31).

However, most studies devoted to AI in healthcare continue to focus on traditional media, policy documents, and academic literature, paying limited attention to the long-term dynamics of hashtag-specific social media discussions and their role in reflecting changing priorities, values, and influence structures in global digital health (32, 33). Analyzing hashtag-tagged discussions over time is a valuable method for identifying changes in topics, tone, and network structures, allowing us to understand how attention and influence are redistributed as technologies evolve and regulatory environments transform (22, 34). In this context, analysing hashtag discussions over time reveals topic frequency and how professional communities negotiate ethical norms, governance, and implementation challenges. Social media platforms, such as X, serve as spaces where clinicians, researchers, policymakers, and patients share their expectations and concerns regarding AI-enabled healthcare. Examining English-language-dominated discourse around #AIinHealthcare reveals how trust, governance, clinical application, and policy are framed during technological change, while acknowledging that this represents hashtag-specific conversations only.

## 2 Methods

### 2.1 Data source and collection

Data were collected using Fedica (https://fedica.com/), a comprehensive social media analytics platform designed for advanced hashtag tracking, audience segmentation and geospatial analysis (35–37). Fedica uses proprietary machine learning for metrics (e.g., geolocation and impressions); no peer-reviewed validation is available; therefore, these are treated as exploratory indicators (see Section 2.1.1 for details on the validation procedure applied). This tool aggregates publicly available tweets from X and provides metrics such as tweet count, impressions, user demographics determined by account geolocation algorithms, types of engagement (likes, retweets, and replies), reach distribution categorised by user follower count, and temporal activity timelines. The analysis concentrated on all tweets containing the hashtag #AIinHealthcare made between 1 November 2022 12:00 PM and 1 November 2025 12:00 PM UTC. Data extraction was performed on 21 November 2025. #AIinHealthcare was selected for its high volume (57,880 tweets over 3 years) and sustained activity post-ChatGPT launch, outperforming alternatives in engagement, making it the most active English-language healthcare-AI hashtag during the study period. This three-year period was deliberately selected to capture the era immediately following the public introduction of large language models (notably ChatGPT on 30 November 2022), which precipitated significant shifts in healthcare AI discussions towards generative applications. Any tweet incorporating the hashtag #AIinHealthcare was included, regardless of the geographical location of the user, language, type of tweet, or classification of the account profile, which could encompass individual users, organisations, media outlets, or research institutions.

#### 2.1.1 Validation of fedica metrics

To address the potential limitations associated with Fedica analytics, we undertook independent manual validation of 89 tweets tagged with #AIinHealthcare, which were downloaded on February 27, 2026. We manually extracted data on user geolocation, follower counts, likes, reposts, and, where available, impression counts, directly from the X platform. Pearson's correlation coefficients were computed to evaluate the concordance between Fedica and manually retrieved values for likes, reposts, and follower counts, with a coefficient (r) greater than 0.90 indicating a very strong agreement. This procedure was conducted to assess potential systematic bias and enhance the reliability of the findings (Supplementary Material). Fedica-derived metrics showed excellent agreement with manual X ground truth across all 89 tweets: Pearson correlations were r = 1.00 for engagement (likes and reposts) and r = 0.999 for followers. Geolocation matched exactly in 94.4% of the cases (potential discrepancies due to private profiles or incomplete bios). These results confirm the high reliability of Fedica metrics despite platform dynamics (Supplementary Table S2).

### 2.2 Measured variables and outcomes

The key metrics included the volume of tweets, encompassing the total number of distinct tweets and shares; user base, defined by the count of unique participants; reach, measured by total impressions indicating the frequency with which tweets were viewed; geographic distribution at both country and city levels; engagement, defined as total interaction counts (likes, retweets, replies, and quote tweets) categorised by type; reach distribution, categorised by the follower count of the original accounts; temporal patterns, examined using monthly tweet volume defined as the number of tweets containing #AIinHealthcare per calendar month; and content themes, identified through commonly co-occurring hashtags and keywords.

### 2.3 Data exclusions and limitations

Following the methodological guidance for social media research, this study excluded demographic attributes such as user gender, occupation, and age. Although Fedica provides demographic segmentation through machine learning algorithms, these classifications lack validated and peer-reviewed methodological documentation. Research has shown that algorithmically inferred demographic attributes may introduce systematic bias, misclassification, and reproducibility issues. To maintain transparency and rigor, we limited our analysis to directly observable platform metrics (e.g., tweet counts, engagement types, co-occurring hashtags, and geolocation), for which we also applied exploratory validation of reliability (as indicated in Section 2.1.1). Analysis focused exclusively on #AIinHealthcare-tagged content, excluding untagged discussions. Other limitations include geographic conclusions limited by lack of user-base normalization and platform access restrictions (e.g., China, Russia).

Ethical considerations followed the frameworks for Internet-mediated and social media research (22). Only public content was analysed; no attempts were made to identify users, access private accounts, or reconstruct profiles. The data were aggregated at the hashtag and population levels, with no tweets quoted in ways that enabled traceability. This approach aligns with ethical recommendations for privacy protection, contextual integrity, data minimization, and harm avoidance in digital research.

### 2.4 Data analysis

The descriptive analysis provided a thorough overview of the dataset. The geospatial analysis clarified the contributions of various countries and cities, and the engagement analysis evaluated different types of interactions. The analysis of hashtag co-occurrence and frequency, complemented by a qualitative review of the top-engagement tweets, was conducted in two stages. In stage 1, frequently co-occurring hashtags and keywords were automatically extracted using Fedica to identify quantitative patterns. In stage 2, a purposive sample of high-engagement tweets (approximately 100 tweets per major cluster) was qualitatively reviewed and inductively coded to validate and refine the thematic clusters and associated stakeholder types.

Sentiment was characterized using Fedica's integrated natural language processing classifier, which categorizes tweets as positive, neutral, or negative. In this context, the neutral category predominantly reflects technical and informational tones. No supplementary manual sentiment annotation or external validation of these labels was conducted; consequently, the sentiment estimates were interpreted as broad, tool-dependent indicators rather than precise psychological measurements. Due to the unavailability of data, normalization by country X-user base was not performed; thus, all metrics are descriptive.

### 2.5 Ethical considerations

This study exclusively employed publicly available data without engaging in active data collection from individuals. The data utilised comprised anonymised social media metrics, ensuring the non-disclosure of personal information. As hashtag analysis involves passive examination of publicly accessible discussions rather than proactive research on human subjects, a formal ethics committee review was deemed unnecessary for this study. Nonetheless, ethical guidelines pertinent to social media research were considered in all analytical decisions.

## 3 Results

### 3.1 Dataset characteristics and volume

In the three-year study period, the hashtag #AIinHealthcare appeared in 57,880 tweets by 17,991 unique users across 141 countries and 1,443 cities, resulting in an estimated 39,237,384 total impressions (Table 1). This suggests that the content associated with this hashtag was viewed approximately 39.2 million times on the X platform. On average, each tweet garnered 678 impressions, and each contributing user generated approximately 2,181 impressions. These metrics indicate that #AIinHealthcare content achieved significant viral reach and high engagement, reflecting widespread and growing interest among diverse global stakeholders.

**Table 1:** Overall summary of Fedica social media analytics on global #AIinHealthcare English-language-dominated discourse on X.

Metric	Value
Total tweets analysed	57,880
Unique users	17,991
Total impressions	39,237,384
Countries	141
Top 3 countries	USA (40.7%), Canada (21%), India (13.4%) = 75.1%
Users with <1,000 followers	74.9% (grassroots)
Total engagements	72,625
Likes (% of engagement)	68.1%
Retweets (% of engagement)	19.4%
Top hashtag	#Healthcare (10,732 co-occurrences)
Primary language	English (96.9%)
Dominant sentiment	Neutral (95%)
Highest engagement	Entrepreneurship (1,198 total engagement)

### 3.2 Geographic distribution

**Figure 1:**
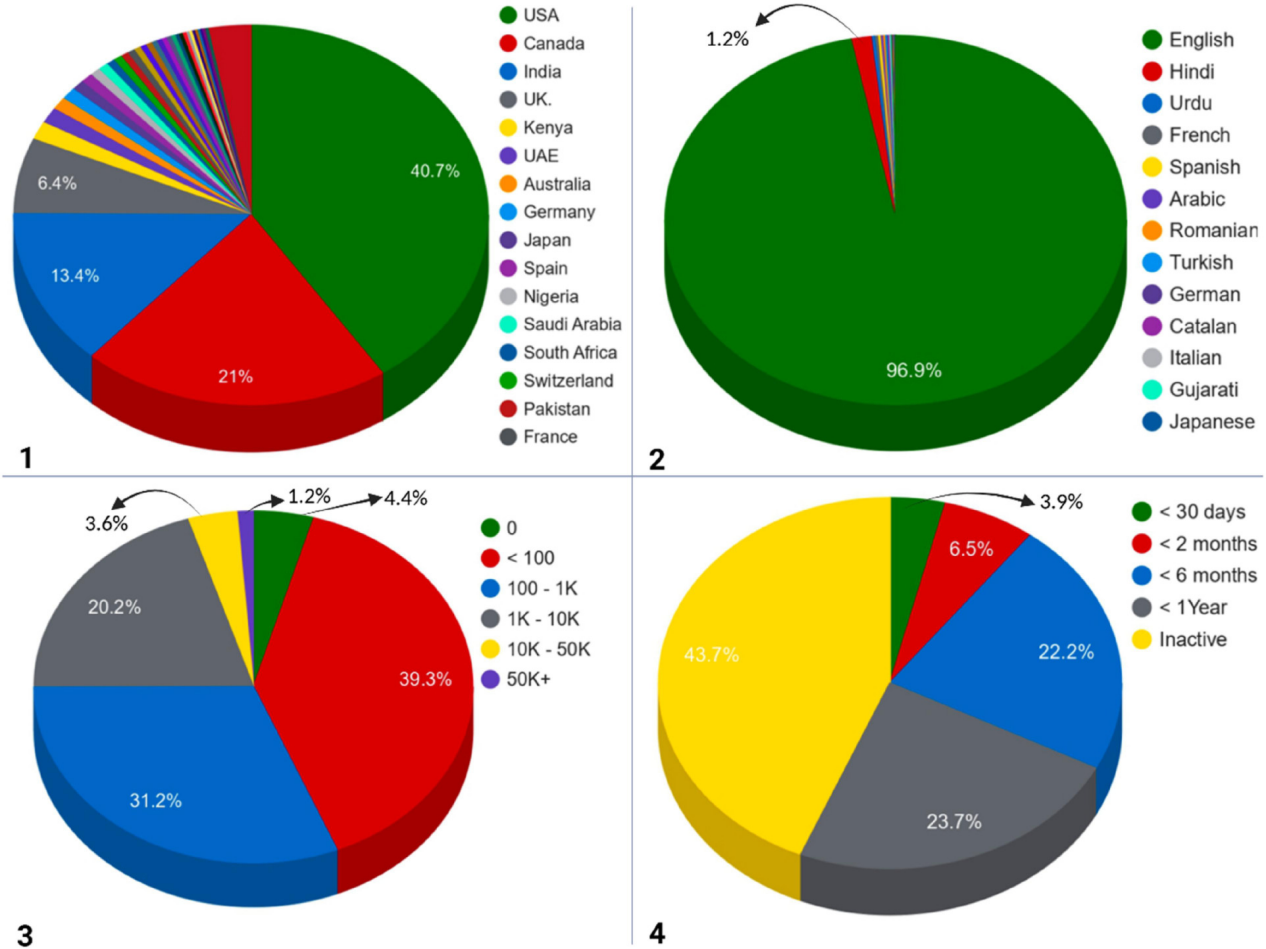
Geographic distribution (**1**), multilingual engagement (**2**), user follower distribution (**3**), and tweet volume (**4**) of #AIinHealthcare English-language-dominated discourse on X based on Fedica social media analytics.

The #AIinHealthcare discourse exhibits a notably concentrated geographic distribution, with the top three countries, the United States, Canada, and India, comprising 75.1% of all tweets (Figure 1). The United States was the predominant contributor, accounting for 23,503 tweets (40.7%). In the United States, the metropolitan areas of Los Angeles, New York City, and Washington, D.C. were identified as key hubs, likely because of the concentration of AI research firms, healthcare technology start-ups, and policy organisations.

Canada ranked second with 12,141 tweets, representing 21% of the total contributions, with Montreal being the most notable city. This is noteworthy given Canada's comparatively smaller population, suggesting a relatively higher per-capita engagement with #AIinHealthcare. The prominence of Montreal highlights its status as a leading global hub for AI research.

India ranked third with 7,767 tweets, which represented 13.4% of the total contributions. This significant outcome underscores the expanding influence of the country in global discourse on digital health innovation. The increase in Indian participation reflects the nation's burgeoning biotech and health-tech sectors and improved digital connectivity, which enables global engagement.

Significant contributions were also made by the United Kingdom (3,719 tweets), Kenya (885 tweets), the United Arab Emirates, Australia, and several European countries. Notably, there was substantial representation of contributors from sub-Saharan Africa, South Asia, and Latin America, indicating a truly global reach.

The country-level shares within the #AIinHealthcare dataset are influenced by platform availability, resulting in limited representation from countries such as China and Russia due to X's restrictions. City-level geolocation data were derived from Fedica's algorithmic inferences and did not represent verified locations. Moreover, normalization by the national X user base was not conducted because of the unavailability of relevant data.

### 3.3 Multilingual engagement

The analysis indicated that 96.9% of tweets were composed in English, reflecting the English-language-dominated orientation of the #AIinHealthcare hashtag rather than the comprehensive global discourse on AI in healthcare; this methodology excludes concurrent non-English discussions occurring under alternative hashtags (Figure 1). Significant contributions also emerged from languages such as Hindi, Urdu, French, Spanish, Arabic, and others. The predominance of English-language contributions, particularly from more affluent nations, underscores the persistent global disparities in digital health engagement on this platform. Notably, the presence of Hindi-language content is significant, given that India contributed 13.4% of the tweet volume. This suggests a level of cultural adaptation and localization within Indian communities, although English remains the predominant language, even in tweets originating from India.

### 3.4 User characteristics and network structure

An analysis of user characteristics revealed that the #AIinHealthcare English-language-dominated discourse was characterised by a diverse and decentralised group of participants rather than being dominated by a limited number of influencers with substantial followings.

#### 3.4.1 Follower distribution

In #AIinHealthcare tweets, 74.9% of users possessed fewer than 1,000 followers, while only 4.8% had over 10,000 followers (Figure 1). The most active contributors were those with follower counts ranging from 100 to 1,000, collectively generated approximately 29,000 tweets. This group was followed by users with fewer than 100 followers, produced approximately 18,000 tweets. Although accounts with over 50,000 followers contributed fewer tweets, they experienced disproportionately high levels of amplification. This distribution suggests that the discourse is predominantly driven by grassroots participation from healthcare professionals, researchers, innovators, and interested members of the public rather than by high-profile celebrity influencers or media outlets, indicating genuine community engagement with the topic.

#### 3.4.2 Temporal patterns

In November 2022, following the public release of ChatGPT, monthly #AIinHealthcare tweet volume increased to approximately 1,200 tweets. Monthly tweet counts peaked in mid-2023 at 1,600–1,800 tweets before declining to 750–900 tweets per month by June 2025. An analysis of user activity revealed that 3.9% of users contributed within the last 30 days (very active), 6.5% tweeted within the past two months (active), and 22.2% were active within the last six months (moderate). Furthermore, 23.7% of users contributed within the past year (less active), indicating that 56.3% of users maintained engagement over a twelve-month period. However, 43.7% of accounts exhibited complete inactivity, suggesting that while a core group sustains ongoing discourse, nearly half of the accounts represent transient or lapsed contributors rather than active participants (Figure 1).

### 3.5 Hashtag co-occurrence and frequency analysis

The analysis of hashtag co-occurrence uncovered a complex and multi-layered discourse structure encompassing five thematically clustered areas (Table 2) in the analyzed dataset (*n* = 23,503 tweets; aligned with US-dominant contributions at 40.7% of the total). Initial discussions were primarily characterized by foundational and technical themes, as evidenced by hashtags such as #GenerativeAI, #LargeLanguageModels, #ChatGPT, #LLMs, #MachineLearning, #DeepLearning, #ComputerVision, and #NeuralNetworks. These hashtags reflected intensive technical benchmarking and model evaluation activities conducted by AI developers and researchers. Clinical application themes revealed disease-specific innovation trajectories, as indicated by hashtags like #CancerCare, #Oncology, #DiseaseDetection, #DiagnosticsAI, #Radiology, #Pathology, #ClinicalTrials, #DrugDiscovery, #MentalHealth, #Stroke, #Cardiology, and #Neurology.

**Table 2:** Top 20 co-occurring hashtags across distinct clusters in the English-language-dominated #AIinHealthcare discourse on X (*n* = 23,503), based on Fedica analytics.

Hashtag co-occurring with #AIinHealthcare	Co-occurrence frequency	Cluster
#Healthcare	10,732	Healthcare
#HealthTech	8,700	Tech
#DigitalHealth	5,584	Tech
#HealthcareInnovation	3,949	Innovation
#ArtificialIntelligence	2,865	Tech
#MedTech	2,744	Tech
#Innovation	1,901	Innovation
#MachineLearning	1,767	Tech
#AIForGood	1,709	Ethics
#PatientCare	1,709	Clinical
#MedicalAI	1,390	Clinical
#FutureOfMedicine	1,297	Clinical
#MedTwitter	1,226	Professional
#GenerativeAI	1,221	Tech
#ResponsibleAI	1,017	Ethics
#DrugDiscovery	1,015	Clinical
#MedicalInnovation	1,012	Innovation
#MentalHealth	630	Clinical
#Telemedicine	603	Clinical
#ChatGPT	504	Tech

Clusters pertaining to the healthcare system and its implementation have emerged as pivotal elements of discourse, with hashtags such as #DigitalHealth, #HealthTech, #Telemedicine, #HealthcareInnovation, #PatientCare, #HealthcareSystems, #EHR, and #MedicalRecords underscoring the practical challenges of integration. Furthermore, the themes of governance and ethics have gained considerable prominence, as evidenced by hashtags such as #ResponsibleAI, #AIEthics, #AIGovernance, #TrustworthyAI, #DataPrivacy, #HealthDataSecurity, and #ExplainableAI. The themes of community and professional engagement, exemplified by hashtags such as #MedTwitter, #MedPulseAI, #HealthcareCareers, and #MedicalEducation, illustrate the development of professional learning communities.

Notably, content associated with entrepreneurship cluster, including startup announcements and health technology innovation, elicited the highest total engagement, with 1,198 interactions, despite a lower frequency of hashtag usage. This suggests significant community interest in the commercial applications of AI in healthcare. The hashtag architecture indicates that discussions on #AIinHealthcare progressed from an initial technical fascination to clinical exploration and, subsequently, to considerations of implementation, governance, and professional integration. This evolution reflects a community increasingly focused on the responsible and practical adoption of AI.

### 3.6 Engagement metrics

**Figure 2:**
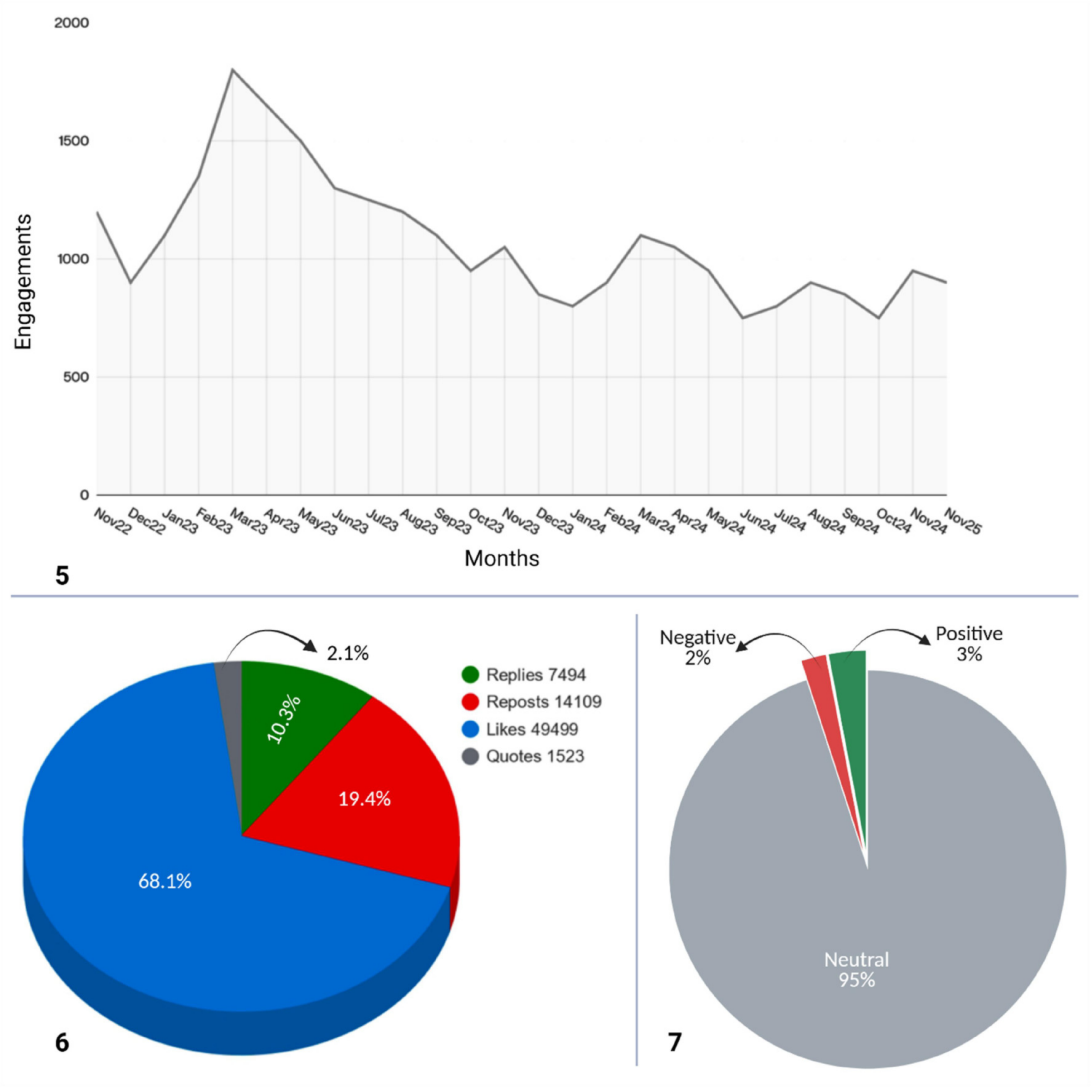
Monthly engagement trends (**5**), engagement distribution (**6**), and sentiment distribution (**7**) of the #AIinHealthcare English-language-dominated discourse on X, based on Fedica social media analytics.

The hashtag #AIinHealthcare garnered 72,625 engagement interactions, indicating significant interest and visibility among X users. Most of this activity was attributed to likes (49,499; 68.1%), suggesting that users primarily engaged with the content in a passive yet supportive manner. This pattern aligns with broader social media trends, where likes remain the most prevalent form of engagement. Retweets accounted for 14,109 actions (19.4%), demonstrating that nearly one-fifth of the engagements contributed to extending the hashtag's reach by disseminating tweets to new user networks. In contrast, replies (7,494; 10.3%) and quote tweets (1,523; 2.1%) represented smaller proportions, indicating that while the topic encourages widespread consumption and endorsement of content, it elicits comparatively limited direct dialogue and evaluative commentary. #AIinHealthcare functions more as a broadcast channel than as an online discussion forum. In general, the engagement pattern suggests a high level of audience interest but a more reserved degree of participatory discussion concerning #AIinHealthcare (Figure 2).

### 3.7 High-engagement contents

The most engaging tweets in the #AIinHealthcare discourse were predominantly influenced by governmental initiatives, academic dialogues, startups, and thought leadership. A notable tweet from the *Viksit Bharat* (developed India) Ambassador program, which underscored #AIinHealthcare as a component of India's vision (*Viksit Bharat*@2047), garnered 441 engagements. Tweets with high engagement included a call for papers by JMIR Publication by Castonguay and Lovis (2023) on AI language models in health, reflecting the scholarly interest in the clinical applications of AI (38). This tweet garnered 940 engagements. Tweets from startups concerning AI-enabled education, healthcare, and an AI clinic assistant for diabetes care attracted interactions by linking AI to practical applications. Research-focused content also attracted strong engagement, with the MedHELM benchmark tweet receiving 412 engagements, underscoring the growing emphasis on evaluating AI models for readiness in real-world clinical tasks. Moreover, tweets from biotech and policy figures regarding AI cancer therapy, health system modernisation, and AI conferences were highly ranked, indicating the community's interest in tweets connecting AI to advancements in cancer care, health equity, and system transformation.

### 3.8 Sentiment and tone

The analysis of the #AIinHealthcare community showed predominantly neutral sentiment, with Fedica's classifier finding 95% neutral tweets, 3% positive, and 2% negative (Figure 2). The high neutral sentiment reflects the professional and informational nature of the discourse, with content focused on research findings, conference announcements, policy updates, product launches, and technical evaluations rather than on opinions. Users typically adopt descriptive language rather than emotional expression.

The hashtag is widely used by clinicians, researchers, institutions, and startups who frame communications cautiously, given the regulatory and ethical concerns in healthcare AI. Low negative sentiment suggests that critical perspectives are presented analytically (discussing bias, validation, governance, or limitations) rather than emotionally, leading to neutral classification. Thus, neutrality reflects both professional community norms and health technology communication patterns on social media.

## 4 Discussion

### 4.1 Geographic significance and global participation

AI technologies are rapidly evolving and being deployed in different geographical locations and across diverse societal domains, including agriculture, finance, education, and public services, with healthcare emerging as one of the most impactful and widely discussed areas (39–41). The geographic distribution of #AIinHealthcare tweets indicates a predominance of high-income countries, with North America accounting for approximately 62% of the total activity. India is emerging as a significant contributor, representing 13.4% of tweets. However, the availability of the platform and censorship issues complicate representation from regions such as China and Russia, where restrictions on X are prevalent. North America, particularly the United States and Canada, exemplifies advanced technological infrastructure, substantial research funding, and a high startup density. This observation is consistent with broader findings that indicate that 74% of AI-enabled clinical research is conducted in high-income settings (42). Another study indicated that the United States and China alone contribute approximately 45% of the global AI clinical research output (43). This situation represents a structural imbalance in which high-income countries dominate AI innovation, infrastructure, and funding, thereby reinforcing digital health leadership in regions with strong research ecosystems and venture capital support (44).

India's 13.4% contribution signifies significant advancement towards a multipolar digital health environment, propelled by rapid progress in health technology innovation and national initiatives such as the India AI Impact Summit 2026. Recent advancements encompass AI-enabled radiology diagnostics, thermal imaging for breast cancer detection, and ophthalmic screening platforms tailored for resource-limited settings, all supported by the *Ayushman Bharat* Digital Mission (formerly the National Digital Health Mission) (45, 46). However, participation from sub-Saharan Africa, Southeast Asia (excluding India), and Central America remains limited, highlighting persistent global disparities exacerbated by infrastructure challenges, such as inadequate broadband, unstable electricity, human resource shortages, and weak regulatory frameworks (22, 24). Models predominantly trained on data from high-income countries risk intensifying inequalities without local adaptation (45).

English predominated (96.9% of tweets), indicating that the #AIinHealthcare hashtag serves as a marker of professional discourse rather than a representation of comprehensive global coverage. A similar bias towards English is evident in studies of related hashtags (#MedTwitterAI, #GlobalHealth, #DataSavesLives), primarily originating from the United States, United Kingdom, Canada, and India (22, 24, 47). Although researchers who do not speak English as their first language strategically employ English to gain international visibility, this linguistic preference conceals parallel discussions occurring in Mandarin, Spanish, French, German, Arabic, and other languages. Consequently, #AIinHealthcare highlights structural disparities in AI readiness, research capacity, and digitization of health systems, underscoring the need for inclusive, multilingual approaches (44, 46).

### 4.2 Hashtag trends and the generative AI transition

The significant increase in #AIinHealthcare activity observed towards the end of 2022 coincides with the public introduction of ChatGPT, marking an interpretive inflection point we refer to as the “generative AI shock”, rather than a formal empirical model. Patterns of hashtag co-occurrence suggest an interpretive narrative of discourse evolution, transitioning from technical benchmarking (#GenerativeAI, #LLMs) to clinical applications (#Oncology, #Radiology), and subsequently to governance concerns (#ResponsibleAI, #AIEthics). Governance-related hashtags experienced a notable rise post 2023, with #ResponsibleAI co-occurrence increasing from 12% of ethics-cluster tweets in 2022 to 28% in 2025 and #AIEthics from 8% to 22% over the same period, according to Fedica temporal data. Similar pivotal moments were evident across the platforms. An analysis of Reddit discussions on AI and mental health revealed a substantial increase post-ChatGPT, with 74% of conversations occurring after November 2022, indicating a shift in focus from rule-based chatbots to large language models (LLMs) (48). Discourse on X regarding cardiology-AI reflects demonstrated efficacy in ECG/image analysis (49), while applications in mental health remain cautious owing to limitations in LLM reliability (50).

Both theoretical and empirical research have identified ChatGPT as a disruptive force in medicine, significantly transforming documentation, education, and patient interaction, while simultaneously raising concerns regarding its reliability, potential for hallucinations, and the necessity for medical oversight (51). The proposed interpretive three-phase narrative (2022-early 2023: technical enthusiasm → mid-2023: clinical specialization → 2024–2025: governance maturation) characterizes the late-2022 #AIinHealthcare surge as a generative AI “shock,” aligning with the literature that documents similar transitions. Following this interpretive inflection, the discourse has shifted from speculative potential to concerns regarding concrete implementation, such as clinical workflows, data pipelines, and decision support. This evolution reflects the consensus that AI should augment, rather than replace, healthcare professionals to maintain the quality of care (52). Meta-analyses of human-AI collaboration confirm superior accuracy and efficiency compared to either entity operating alone (53).

The increasing prominence of #ResponsibleAI, #AIEthics, and #ExplainableAI within the #AIinHealthcare English-language-dominated discourse reflects a shift in the literature from an initial focus on innovation optimism to concerns regarding implementation and governance. Systematic reviews of generative AI in healthcare underscore persistent challenges, such as systemic bias arising from non-representative data, opaque “black box” decision-making processes, ambiguous legal liability, and privacy risks, thereby advocating for technical solutions to be accompanied by robust regulatory oversight (54). Disease-specific tags (#Oncology, #Cardiology) indicate a scientific trend towards domain-specialized AI systems that necessitate thorough clinical validation, context-aware implementation, and alignment with ethical and legal standards, rather than generic solutions (45, 54). Efforts to bridge the gap between algorithm development and clinical deployment emphasize the importance of data quality, interoperability, and multidisciplinary governance (55).

The heightened engagement of communities with regulatory matters highlights the significance of the EU AI Act, which introduces the world's first comprehensive requirements for high-risk healthcare AI systems (56, 57). Scholars have advocated the integration of AI ethics and governance into medical curricula to adequately prepare clinicians for the ethical, legal, and societal implications of AI (58). Collectively, these developments support our narrative of interpretive governance maturation: a progression from technical enthusiasm to clinical applications and, ultimately, to governance priorities, emphasizing safety, accountability, and clinical relevance for the sustainable integration of AI.

### 4.3 Stakeholder diversity and the bridging function of X

The #AIinHealthcare hashtag serves as a unique conduit for uniting traditionally distinct groups, including academic researchers, clinicians, health technology entrepreneurs, venture capitalists, policymakers, journalists, and the general public. This phenomenon reflects a broader trend in research that recognises social media as a platform for interdisciplinary interactions. Traditional methods of knowledge dissemination, such as peer-reviewed journals and professional conferences, often uphold disciplinary boundaries, thereby limiting the exchange between evidence creators and users (59). In contrast, the open and conversational nature of X offers what Campbell et al. (2024) describe as “rapid access to succinct knowledge” and “networking opportunities” that transcend institutional constraints (59). A study involving first-contact practitioners revealed that X mitigates professional isolation by facilitating dialogue, sharing best practices, and providing access to evidence that is frequently obscured by paywalls (59). This supports the notion that #AIinHealthcare tweets can simultaneously inform researchers, engage investors, educate clinicians, and stimulate policy discussions, thereby effectively streamlining multiple knowledge translation processes into a single publicly accessible thread.

Empirical investigations of analogous hashtag-driven campaigns further substantiate this bridging function. A study demonstrated that the #MedTwitterAI campaign effectively engaged a geographically diverse group of healthcare stakeholders, including clinicians, patient advocates, caregivers, researchers, and journalists, by curating content at the intersection of computer science and medicine (22). The authors attribute this extensive participation to X's cost-effective, wide-reaching capabilities and its function as a “virtual space” where diverse participants collaboratively construct narratives about AI applications. Similarly, another study examined stroke-related discussions on X and found that tweets were generated by individuals, physicians, media outlets, and health organisations, with tweets from doctors inciting the most conversation despite individual tweets achieving a broader reach (60). This pattern is akin to the #AIinHealthcare ecosystem, where accounts with fewer than 100 followers contribute numerous tweets, yet high-engagement content frequently originates from verified clinical or policy voices, thereby fostering dynamic interactions between grassroots discussions and authoritative commentaries.

The multi-stakeholder dialogue facilitated by X faces several challenges. Interdisciplinary discussions may result in misunderstandings when technical terminology, regulatory nuances, or clinical contexts are not accurately communicated. A study observed that social media platforms employed for designing health services and enhancing quality often lack adequate management and moderation, leading to concerns about power imbalances and the quality of participant input (61). Nevertheless, the same authors argue that these platforms offer “new ways to engage more diverse stakeholders” and can expedite innovation by uncovering practical obstacles and solutions that might remain unnoticed in traditional settings (60). This balance between the risk of miscommunication and the benefit of rapid, cross-boundary problem-solving characterizes the bridging role of #AIinHealthcare and underscores its significance as a unique convergence point for digital health innovation.

### 4.4 Limitations and methodological considerations

Despite these contributions, this study has several notable limitations. The analysis was predominantly of English-language tweets (96.9%) on platform X that included only the #AIinHealthcare. While this approach ensured analytical consistency, it introduced selection bias by excluding alternative hashtags (#MedAI, #HealthTechAI, regional variants), untagged content, parallel non-English discourses (such as those in Mandarin, Spanish, Arabic, and Portuguese under different hashtags), and other platforms (such as LinkedIn, Weibo, and ResearchGate). All key metrics, geolocation, impressions (39.2 M), engagement, and sentiment (95% neutral) were derived from Fedica's proprietary machine learning algorithms, which lack peer-reviewed validation. City-level locations are based on algorithmic inference rather than verified data, and sentiment classification reflects coarse granularity rather than a nuanced tone. The study employs purely descriptive methods without the use of inferential statistics, hypothesis testing, normalization by country-level X penetration/user base, or formal changepoint analysis; thus, temporal trends capture only proportional hashtag shifts. User demographics (gender, age, occupation) were deliberately excluded due to unreliable machine learning inference, and platform access barriers (X is blocked in China, filtered in Russia, and limited in much of Africa and Latin America) systematically confound geographic representation, leading to an overstatement of the dominance of high-income countries.

Future research should prioritize the following directions: (1) the development of multi-platform designs that encompass LinkedIn, Weibo, and regional social media; (2) the implementation of multi-lingual, multi-hashtag methodologies incorporating automated translation and topic modelling; (3) the execution of normalized geographic analyses that integrate country-level X penetration data; (4) the establishment of validated natural language processing (NLP) pipelines with manual annotation of sentiment, themes, and geolocation; and (5) the conduct of mixed-methods longitudinal studies that combine quantitative network analysis with qualitative content coding of high-impact posts. These extensions are anticipated to provide more comprehensive and representative insights into the global discourse on AI in healthcare.

### 4.5 Broader implications for healthcare innovation communication

This analysis illustrates that innovation in healthcare AI is influenced not only by laboratory and clinical environments but also by digitally mediated public discourse. The activity on X increasingly affects visibility, funding priorities, regulatory focus, and professional legitimacy. The prominence of hashtags such as #ResponsibleAI and #AIEthics parallels significant regulatory developments, including the EU AI Act (2024), US Executive Order 14110 (2023), and India's updated National AI Strategy (2024), positioning social media as both a barometer and accelerant of governance debates. The coexistence of celebratory narratives and governance-oriented concerns reflects what has been described as “cautious optimism” toward medical AI (33), which aligns with calls for responsible innovation, transparency, equity, and accountability (6, 10, 54). However, critical gaps remain. Sustainability remains marginal (<0.5% hashtag co-occurrence), workforce training is underrepresented (1.2% of tweets), and equity-related hashtags account for <3%, raising concerns that digital enthusiasm may outpace practical integration capacity (10, 55). Geographic imbalances further underscore structural divides, with India contributing 13.4% of tweets, while sub-Saharan Africa and Central America together account for <2%. Avoiding “uncritical enthusiasm” therefore necessitates moving beyond innovation narratives toward sustained engagement with scalability, workforce reskilling, sustainability, and equitable access. Monitoring hashtag ecosystems may serve as an early indicator of emerging policy blind spots, complementing traditional health governance and horizon scanning approaches.

## 5 Conclusion

The longitudinal analysis of #AIinHealthcare discourse on X reveals an evolving global conversation driven by English-language contributions from high-income countries and India. The discourse has progressed through three phases: initial enthusiasm for generative AI and language models, exploration of clinical applications, and maturation toward governance and responsible AI deployment. This evolution reflects a focus on practical integration, regulatory compliance, and collaboration. While the platform connects diverse stakeholders, including researchers, clinicians, policymakers, and entrepreneurs, geographic and linguistic disparities highlight the challenges in achieving inclusive global innovation. The predominance of neutral sentiment and grassroots participation shows a professional dialogue that balances optimism with critical reflection. Gaps in sustainability, workforce training, and equitable access suggest areas for future research. The #AIinHealthcare movement serves as both an indicator of technological shifts and a catalyst for knowledge translation, emphasizing the need for continued engagement to support responsible AI adoption in healthcare.

## Data Availability

The original contributions presented in the study are included in the article/Supplementary Material, further inquiries can be directed to the corresponding author/s.
