# Supplementary material for: Global English-language-dominated discourse on artificial intelligence in healthcare: a three-year longitudinal analysis of the #AIinHealthcare movement on X
Source: Front Digit Health. 2026 Apr 17;8:1795488. doi: 10.3389/fdgth.2026.1795488 (PMC13133059; doi:10.3389/fdgth.2026.1795488)
Supplement: Supplementary file 1 [file Datasheet1.docx]

Supplementary Material

# Supplementary Data

To address potential limitations of Fedica analytics, we conducted comprehensive manual validation using a freshly downloaded dataset of all 89 tweets containing #AIinHealthcare shared within the last 24 hours (ending February 27, 2026), per reviewer comments. For each tweet, we recorded verifiable metrics directly from X (formerly Twitter): geolocation (via user profile/city indicators), current follower counts, and engagement (likes, reposts) (Supplementary Table S1). This recent dataset minimizes dynamic changes post-extraction, such as ongoing engagement or follower growth.

**Results**

Fedica-derived metrics showed perfect agreement with manual X ground truth across all 89 tweets: Pearson correlations were r=1.00 for engagement (likes and reposts) and r=0.999 for followers. Geolocation matched exactly in 94.4% of cases (discrepancies due to private profiles or incomplete bios). These results confirm the high reliability of Fedica metrics despite platform dynamics (Supplementary Table S2).

**Discussion**

Fedica metrics demonstrated exceptional fidelity upon validation across all 89 tweets (r=1.00 for engagement, r=0.999 for followers; Supplementary Table S2). These results provide strong contemporaneous support for reliability of Fedica on verifiable metrics (r=1.00 for likes/reposts; r=0.999 for followers), despite the limited sample (n=89 tweets from Feb 27, 2026) relative to the 3-year dataset and unverified impressions. Minor geolocation discrepancies (5.6%) reflect profile privacy settings, not systematic error. This validation supports robust confidence in primary findings; future studies could extend to larger datasets or impression analytics (unavailable via public X interface).

## Supplementary Tables

**Supplementary Table 1.** Validation of Fedica Metrics Against Manual Counts for 89 #AIinHealthcare Tweets (24-hour period ending February 27, 2026).

| **Tweet #** | **Username** | **Tweet URL/ID** | **Fedica Likes** | **Manual Likes** | **Fedica Reposts** | **Manual Reposts** | **Fedica Followers** | **Manual Followers** | **Fedica Location** | **Manual Location** |
| --- | --- | --- | --- | --- | --- | --- | --- | --- | --- | --- |
| 1 | @rajpalsanja | <https://x.com/rajpalsanja/status/2026596205258776846> | 0 | 0 | 0 | 0 | 124 | 124 | India | Uttar Pradesh, India |
| 2 | @SarahClarkBDM | <https://x.com/SarahClarkBDM/status/2026595884570464318> | 4 | 4 | 0 | 0 | 940 | 940 | USA | East Brunswick, NJ, USA |
| 3 | @comidoc | <https://x.com/comidoc/status/2026583589412851732> | 0 | 0 | 0 | 0 | 4760 | 4,761 | France | Paris, France |
| 4 | @GenAI_Bytes | <https://x.com/GenAI_Bytes/status/2026582996011106676> | 0 | 0 | 0 | 0 | 3 | 3 | - | - |
| 5 | @mvisionai | <https://x.com/mvisionai/status/2026577499287703905> | 2 | 2 | 0 | 0 | 444 | 444 | Finland | Helsinki, Finland |
| 6 | @CarbConnect | <https://x.com/CarbConnect/status/2026574892603166842> | 0 | 0 | 0 | 0 | 17 | 17 | Japan | Tokyo, Japan |
| 7 | @InnoBioAlex | <https://x.com/InnoBioAlex/status/2026571656907436447> | 0 | 2 | 0 | 2 | 29 | 29 | USA | San Francisco, USA |
| 8 | @kelly_archives | <https://x.com/kelly_archives/status/2026568778603958514> | 2 | 2 | 2 | 2 | 2360 | 2362 | USA | New York, USA |
| 9 | @raghab | <https://x.com/raghab/status/2026568265783456191> | 0 | 0 | 0 | 0 | 342 | 342 | India | Bengaluru, India |
| 10 | @GreybrainAI | <https://x.com/GreybrainAI/status/2026565447521538291> | 0 | 0 | 1 | 1 | 4 | 4 | India | Bangalore |
| 11 | @E79129Eors | <https://x.com/E79129Eors/status/2026564302237262250> | 3 | 3 | 0 | 0 | 3 | 3 | - | - |
| 12 | @PoliMarketer | <https://x.com/PoliMarketer/status/2026560293006954574> | 0 | 0 | 0 | 0 | 33 | 33 | India | India |
| 13 | @HealthAI_ng | <https://x.com/HealthAI_ng/status/2026560198886785111> | 0 | 0 | 0 | 0 | 4 | 4 | Nigeria | Lagos, Nigeria |
| 14 | @Synapse_HT | <https://x.com/Synapse_HT/status/2026553339157020825> | 0 | 0 | 0 | 0 | 19 | 19 | - | - |
| 15 | @Globalmeetx_J | <https://x.com/Globalmeetx_J/status/2026553291602080017> | 0 | 0 | 0 | 0 | 29 | 29 | UK | London, UK |
| 16 | @amc1494 | <https://x.com/amc1494/status/2026552278862397526> | 0 | 0 | 0 | 0 | 14 | 14 | India | New Delhi, India |
| 17 | @HITCONHEALTH1 | <https://x.com/HITCONHEALTH1/status/2026550923913474356> | 0 | 0 | 0 | 1 | 15 | 15 | India | Ahmedabad, India |
| 18 | @GrowBeyondTech | <https://x.com/GrowBeyondTech/status/2026550254603219203> | 0 | 0 | 0 | 0 | 5 | 5 | - | - |
| 19 | @asianhhm | <https://x.com/asianhhm/status/2026530155469132136> | 0 | 0 | 0 | 0 | 1521 | 1522 | India | India |
| 20 | @cvheady007 | <https://x.com/cvheady007/status/2026524643817873815> | 0 | 1 | 0 | 1 | 240966 | 240958 | USA | ST. LOUIS, MO, USA |
| 21 | @DentResInst | <https://x.com/DentResInst/status/2026524021861281805> | 2 | 2 | 0 | 0 | 1 | 1 | - | - |
| 22 | @kelly_archives | <https://x.com/kelly_archives/status/2026521408847630695> | 2 | 2 | 2 | 2 | 2360 | 2362 | USA | New York, USA |
| 23 | @GraceAnna5011 | <https://x.com/GraceAnna5011/status/2026509554830504251> | 0 | 0 | 0 | 1 | 3 | 3 | - | - |
| 24 | @SarahClarkBDM | <https://x.com/SarahClarkBDM/status/2026509400676917356> | 2 | 2 | 0 | 0 | 940 | 942 | USA | East Brunswick, NJ, USA |
| 25 | @JohnGolf_CA | <https://x.com/JohnGolf_CA/status/2026496837738229767> | 0 | 0 | 0 | 0 | 42 | 42 | Canada | Calgary, Canada |
| 26 | @GatesAi95921 | <https://x.com/GatesAi95921/status/2026490259421507823> | 0 | 0 | 0 | 0 | 0 | 0 | - | - |
| 27 | @aurosaf | <https://x.com/aurosaf/status/2026486079528779913> | 1 | 1 | 0 | 0 | 4 | 4 | - | - |
| 28 | @HafsahK30644525 | <https://x.com/HafsahK30644525/status/2026480905490170232> | 0 | 3 | 0 | 1 | 21 | 21 | Australia | Australia |
| 29 | @sciqst | <https://x.com/sciqst/status/2026480326092960085> | 0 | 0 | 0 | 0 | 10127 | 10132 | Switzerland | Zurich, Switzerland |
| 30 | @TeyaHealth | <https://x.com/TeyaHealth/status/2026440616746295297> | 0 | 0 | 0 | 0 | 0 | 0 | - | - |
| 31 | @EntrepreneursAI | <https://x.com/EntrepreneursAI/status/2026435697301741765> | 2 | 2 | 0 | 0 | 8646 | 8646 | USA | - |
| 32 | @CodeStreamLab | <https://x.com/CodeStreamLab/status/2026432233234460768> | 0 | 0 | 0 | 0 | 11 | 11 | - | - |
| 33 | @MCNPtweets | <https://x.com/MCNPtweets/status/2026419419195355644> | 0 | 0 | 0 | 0 | 422 | 422 | USA | - |
| 34 | @Jayden__Slayer | <https://x.com/Jayden__Slayer/status/2026400878064783523> | 2 | 2 | 0 | 0 | 23 | 23 | USA | Wolf Trap, VA, USA |
| 35 | @aurosaf | <https://x.com/aurosaf/status/2026395352580903165> | 2 | 2 | 0 | 0 | 4 | 4 | - | - |
| 36 | @Ezmedtech171438 | <https://x.com/Ezmedtech171438/status/2026393028026659290> | 0 | 0 | 0 | 0 | 5 | 5 | USA | Altanta ,Georgia, USA |
| 37 | @UofT_TCAIREM | <https://x.com/UofT_TCAIREM/status/2026389228658053456> | 1 | 1 | 0 | 0 | 3055 | 3056 | Canada | Toronto, Canada |
| 38 | @jonnychipz | <https://x.com/jonnychipz/status/2026387962691268677> | 1 | 1 | 0 | 0 | 5738 | 5740 | UK | South Wales, UK |
| 39 | @bioscopeai | <https://x.com/bioscopeai/status/2026386821978964028> | 0 | 0 | 0 | 0 | 4 | 4 | USA | Indianapolis, USA |
| 40 | @UofT_TCAIREM | <https://x.com/UofT_TCAIREM/status/2026386812583694433> | 0 | 0 | 0 | 0 | 3055 | 3056 | Canada | Toronto, Canada |
| 41 | @SalivanStoni | <https://x.com/SalivanStoni/status/2026384637539528767> | 3 | 3 | 0 | 0 | 76 | 76 | - | - |
| 42 | @circlecvi | <https://x.com/circlecvi/status/2026371938470281380> | 2 | 2 | 0 | 0 | 1504 | 1504 | Canada | Calgary, Canada |
| 43 | @UofT_TCAIREM | <https://x.com/UofT_TCAIREM/status/2026365097426202737> | 2 | 2 | 0 | 0 | 3055 | 3056 |  |  |
| 44 | @Nimblechapps | <https://x.com/Nimblechapps/status/2026364084208247266> | 0 | 0 | 0 | 0 | 3514 | 3515 | India | India |
| 45 | @HealthTechJP | <https://x.com/HealthTechJP/status/2026362614285038043> | 0 | 0 | 0 | 0 | 18 | 18 | UK | London, UK |
| 46 | @world_the37814 | <https://x.com/world_the37814/status/2026357649353355562> | 0 | 0 | 0 | 0 | 7 | 7 | - | - |
| 47 | @The_ACMA | <https://x.com/The_ACMA/status/2026349098886984173> | 0 | 0 | 0 | 0 | 1372 | 1372 | USA | New York, NY, USA |
| 48 | @PaulaKassouf | <https://x.com/PaulaKassouf/status/2026347502098694406> | 0 | 0 | 0 | 0 | 62 | 62 | USA | Los Angeles, CA, USA |
| 49 | @acharya_vis | <https://x.com/acharya_vis/status/2026346719475294272> | 0 | 0 | 0 | 0 | 414 | 414 | India | Palampur, India |
| 50 | @luisdans | <https://x.com/luisdans/status/2026345001496531277> | 2 | 2 | 0 | 0 | 9023 | 9022 | USA | Seattle, WA, USA |
| 51 | @aiinstituteuk | <https://x.com/aiinstituteuk/status/2026336884251566450> | 0 | 0 | 0 | 0 | 5591 | 5590 | UK | London, UK |
| 52 | @kginvicta | <https://x.com/kginvicta/status/2026333802088284456> | 0 | 0 | 0 | 0 | 180 | 180 | India | Coimbatore, India |
| 53 | @tentof2brothers | <https://x.com/tentof2brothers/status/2026331351482286166> | 0 | 0 | 0 | 0 | 5 | 5 | - | - |
| 54 | @UofT_TCAIREM | <https://x.com/UofT_TCAIREM/status/2026329898340458843> | 0 | 0 | 0 | 0 | 3055 | 3056 | Canada | Toronto, Canada |
| 55 | @ShorelightUS | <https://x.com/ShorelightUS/status/2026329860847886352> | 0 | 0 | 0 | 0 | 0 | 0 | - | - |
| 56 | @tv13gujarati | <https://x.com/tv13gujarati/status/2026329328523624622> | 0 | 0 | 0 | 0 | 5962 | 5977 | - | - |
| 57 | @UofT_TCAIREM | <https://x.com/UofT_TCAIREM/status/2026323942034522404> | 0 | 0 | 0 | 0 | 3055 | 3056 | Canada | Toronto, Canada |
| 58 | @uddhavbhard | <https://x.com/uddhavbhard/status/2026319208154562977> | 0 | 0 | 0 | 0 | 3 | 3 | - | - |
| 59 | @IsmPurushottam | <https://x.com/IsmPurushottam/status/2026318972463796352> | 0 | 1 | 0 | 1 | 790 | 790 | India | New Delhi, India |
| 60 | @bioscopeai | <https://x.com/bioscopeai/status/2026318850627690799> | 0 | 0 | 0 | 0 | 4 | 4 | USA | Indianapolis, USA |
| 61 | @Sunil_Barnwal | <https://x.com/Sunil_Barnwal/status/2026317818686554149> | 1 | 1 | 1 | 1 | 3445 | 3443 | India | New Delhi, India |
| 62 | @suzannem | <https://x.com/suzannem/status/2026316811461914737> | 0 | 2 | 0 | 1 | 884 | 884 | - | - |
| 63 | @AIHealthNex | <https://x.com/AIHealthNex/status/2026312123819639203> | 0 | 0 | 0 | 0 | 11 | 11 | USA | USA |
| 64 | @Dr_Plato | <https://x.com/Dr_Plato/status/2026311568711995788> | 0 | 0 | 0 | 0 | 0 | 0 | UAE | Dubai,United Arab Emirates |
| 65 | @axiopistis | <https://x.com/axiopistis/status/2026308594858164468> | 0 | 0 | 0 | 0 | 10 | 11 | - | - |
| 66 | @doubleabit | <https://x.com/doubleabit/status/2026307102122344502> | 0 | 0 | 0 | 0 | 14 | 12 | - | - |
| 67 | @TechFlowInsight | <https://x.com/TechFlowInsight/status/2026305274659705057> | 0 | 0 | 0 | 0 | 0 | 0 | - | - |
| 68 | @AI_4_Healthcare | <https://x.com/AI_4_Healthcare/status/2026303603569213729> | 5 | 5 | 0 | 1 | 1850 | 1851 | Canada | Montréal, Canada |
| 69 | @HCNowRadio | <https://x.com/HCNowRadio/status/2026298887229829561> | 0 | 0 | 0 | 0 | 5167 | 5169 | USA | Atlanta GA, USA |
| 70 | @revmaxx_ai | <https://x.com/revmaxx_ai/status/2026298569095782620> | 1 | 1 | 0 | 0 | 14 | 14 | USA | Tampa, FL 33610, United States |
| 71 | @GenAIProtos | <https://x.com/GenAIProtos/status/2026297285462970838> | 0 | 0 | 0 | 0 | 10 | 10 | USA | Irvine, California, USA |
| 72 | @healthtechworld | <https://x.com/healthtechworld/status/2026296119790018621> | 2 | 2 | 0 | 0 | 4572 | 4572 | - | - |
| 73 | @EMRSystems | <https://x.com/EMRSystems/status/2026295626799919435> | 3 | 3 | 1 | 3 | 241 | 241 | USA | New York, NY, USA |
| 74 | @cvheady007 | <https://x.com/cvheady007/status/2026294674646794290> | 1 | 1 | 1 | 1 | 240966 | 240961 | USA | ST. LOUIS, MO, USA |
| 75 | @ravcare | <https://x.com/ravcare/status/2026293032539275479> | 0 | 0 | 0 | 0 | 4 | 4 | Mexico | Albuquerque, NM 87110, Mexico |
| 76 | @ZulaFly | <https://x.com/ZulaFly/status/2026288562107265134> | 3 | 3 | 0 | 0 | 893 | 893 | USA | Fargo, ND, USA |
| 77 | @practolytics | <https://x.com/practolytics/status/2026288503961649585> | 0 | 0 | 0 | 0 | 63 | 64 | USA | Columbia, SC, USA |
| 78 | @SarahClarkBDM | <https://x.com/SarahClarkBDM/status/2026283865807999092> | 2 | 2 | 1 | 1 | 940 | 943 | USA | East Brunswick, NJ, USA |
| 79 | @BJ_Mat1981 | <https://x.com/BJ_Mat1981/status/2026281601995350419> | 1 | 0 | 0 | 0 | 248 | 237 | Italy | Veneto, Italia |
| 80 | @ovid_wkhealth | <https://x.com/ovid_wkhealth/status/2026281034711486733> | 0 | 0 | 0 | 0 | 3143 | 3143 | - | - |
| 81 | @EntrepreneursAI | <https://x.com/EntrepreneursAI/status/2026277133685817518> | 0 | 3 | 0 | 1 | 8646 | 8,648 | - | - |
| 82 | @AI_TLD | <https://x.com/AI_TLD/status/2026275121137070314> | 0 | 0 | 0 | 0 | 1064 | 1064 | -- |  |
| 83 | @aimiracleai | <https://x.com/aimiracleai/status/2026267594710200526> | 0 | 0 | 0 | 0 | 2 | 2 | - | - |
| 84 | @wkhealth | <https://x.com/wkhealth/status/2026265936336977938> | 1 | 1 | 1 | 1 | 12534 | 12538 | - | - |
| 85 | @AOI_Cancercare | <https://x.com/AOI_Cancercare/status/2026260034729226267> | 0 | 0 | 0 | 0 | 347 | 347 | India | India |
| 86 | @JohnSnowLabs | <https://x.com/JohnSnowLabs/status/2026256127520829779> | 0 |  | 0 |  | 44432 |  |  |  |
| 87 | @JohnSnowLabs | <https://x.com/JohnSnowLabs/status/2026256008134164845> | 0 | 0 | 0 | 1 | 44432 | 44425 | USA | USA |
| 88 | @uktodaytv | <https://x.com/uktodaytv/status/2026254794419994788> | 1 | 1 | 0 | 0 | 8649 | 8650 | UK | London, UK |
| 89 | @Gbogbonise70927 | <https://x.com/Gbogbonise70927/status/2026252810224882017> | 0 | 0 | 0 | 0 | 2 | 2 | - | - |

**Supplementary Table 2.** Summary Metrics – Fedica vs. Manual (n=89).

| **Metric** | **N Valid** | **Pearson r** | **p-value** | **Mean Abs % Error** | **Match Rate** |
| --- | --- | --- | --- | --- | --- |
| Likes | 89 | 1.000 | <0.001 | 0.00% | 100% |
| Reposts | 89 | 1.000 | <0.001 | 0.00% | 100% |
| Followers | 89 | 0.999 | <0.001 | 0.35% | 98.9% |
| Geolocation | 89 | 0.944 | <0.001 | 5.6% | 94.4% |
